# High level of agreement in a fixed vs. live cell-based assay for antibodies to myelin oligodendrocyte glycoprotein in a real-world clinical laboratory setting

**DOI:** 10.3389/fneur.2023.1192644

**Published:** 2023-07-12

**Authors:** Tammy L. Smith, Thomas R. Haven, Lauren M. Zuromski, Kyphuong Luong, Stacey L. Clardy, Lisa K. Peterson

**Affiliations:** ^1^Geriatric Research Education and Clinical Center, George E. Whalen Department of Veterans Affairs Medical Center, Salt Lake City, UT, United States; ^2^Department of Neurology, University of Utah School of Medicine, Salt Lake City, UT, United States; ^3^ARUP Institute for Clinical and Experimental Pathology, Salt Lake City, UT, United States; ^4^Department of Pathology, University of Utah, Salt Lake City, UT, United States; ^5^Neurology Service, George E. Whalen Department of Veterans Affairs Medical Center, Salt Lake City, UT, United States

**Keywords:** myelin oligodendrocyte glycoprotein (MOG), cell-based assay (CBA), MOG testing, myelin oligodendrocyte glycoprotein antibody-associated disease (MOGAD), demyelinating disease, fixed CBA, live CBA

## Abstract

**Introduction:**

As recognition of myelin oligodendrocyte glycoprotein (MOG) antibody-associated disease becomes more widespread, the importance of appropriately ordering and interpreting diagnostic testing for this antibody increases. Several assays are commercially available for MOG testing, and based on a few small studies with very few discrepant results, some have suggested that live cell-based assays (CBA) are superior to fixed CBA for clinical MOG antibody testing. We aimed to determine the real-world agreement between a fixed and live CBA for MOG using two of the most commonly available commercial testing platforms.

**Methods:**

We compared paired clinical samples tested at two national clinical reference laboratories and determined the real-world agreement between the fixed CBA and live CBA.

**Results:**

Of 322 paired samples tested on both platforms, 53 were positive and 246 were negative by both methodologies (agreement 92.9%, Cohen’s kappa 0.78, [0.69-0.86]). Spearman correlation coefficient was 0.80 (*p* < 0.0001). Of the discrepant results, only 1 of 14 results positive by the live CBA had a titer greater than 1:100, and only 1 of 9 results positive by the fixed CBA had a titer of greater than 1:80. Lower titers on the fixed CBA correlate to higher titers on the live CBA.

**Conclusion:**

Overall, there is excellent agreement between fixed and live CBA for MOG antibody testing in a real-world clinical laboratory setting. Clinicians should be aware of which method they use to assess any given patient, as titers are comparable, but not identical between the assays.

## Introduction

Clinical testing for antibodies to myelin oligodendrocyte glycoprotein (MOG) is increasingly done in the workup of suspected immune-mediated inflammatory demyelinating disorders of the central nervous system, given the phenotypic overlap of MOG antibody-associated disease (MOGAD) with neuromyelitis optica (NMO) spectrum disorders and multiple sclerosis (MS). In adults, MOGAD most often presents with optic neuritis, myelitis, or a combination of the two (1). In pediatric patients, the initial presentation is most commonly acute disseminated encephalomyelitis (ADEM) or optic neuritis (2–4). When compared to those who are positive for antibodies to aquaporin-4 (AQP4), MOGAD patients tend to be younger, with equal numbers of males and females affected, and are more likely to have a monophasic disease course (5–10). The recognition that MOGAD represents a unique clinical syndrome with distinct epidemiology, relapse rates, and treatment responses has led to guidelines for diagnosis and antibody testing (11, 12).

As antibody testing for MOG becomes more widespread, it is critical that clinicians understand the differences between the various assays. Early studies of serum MOG antibodies used western blot or enzyme-linked immunosorbent assay (ELISA) to detect the presence of autoantibodies; these assays showed that up to 38% of MS patients and 53% of patients with other inflammatory neurologic diseases (viral or bacterial encephalitis) have detectable MOG IgG, compared to 3% in patients with noninflammatory neurologic diseases (13). However, when cell-based assays (CBAs) using cell lines expressing native proteins were subsequently developed to detect anti-MOG antibodies, they were able to more clearly distinguish MS patients (negative for MOG-IgG with nearly 100% specificity) from MOGAD patients (14, 15). The technical differences between assays which use peptide antigens or denatured proteins (e.g., western blot, ELISA) and those using native full-length proteins (e.g., CBAs) are important to consider when interpreting test results; currently, CBAs are recommended for the diagnosis of MOGAD (12, 16, 17).

When CBAs are used for detection of MOG-IgG, laboratories may use live CBAs with detection via immunofluorescence (CBA-IF) or flow cytometry (CBA-FC) or fixed CBAs with detection via IF (fCBA-IF). Fixed CBAs are widely used in diagnostic laboratories because they allow for the purchase of validated, prepared slides from commercial sources. Live CBAs require maintenance and validation of the cell line within the individual laboratory, a technical hurdle, which limits their utility outside of specialized laboratory settings. Studies comparing these CBAs at multiple institutions using sera from patients with clinically defined syndromes or previously defined seropositive or seronegative samples have led to the dogma that live CBA are superior to fixed CBA (18–20). In this study, we sought to determine the real-world agreement between live CBA-FC and fCBA-IF performed at two major clinical reference laboratories in the United States using samples sent in for routine clinical testing.

## Materials and methods

### Standard protocol approvals, registrations, and patient consents

This study was approved by the University of Utah IRB (IRB_0082990 for the retrospective analysis and IRB_00068933 for the validation and prospective testing); participant consent was waived, as data were extracted using a limited dataset and all testing was performed on residual clinical samples. Patient sera were identified by retrospective analysis of results from samples received from United States medical centers and tested for MOG antibodies between February 2019 and November 2022 at both ARUP Laboratories and Mayo Clinic Laboratories (MCL). Patients were included if they had MOG antibody testing performed at both ARUP and MCL using serum collected within 30 days of one another (to limit the possible impact of treatment on antibody titer). If multiple serum pairs from a given patient were available, the earliest submitted samples were prioritized, followed by the samples with the smallest time difference between them, for analysis. Patients were excluded if they had a MOG antibody result from only one of the laboratories, or multiple results from the same patient using sera collected more than 30 days apart. In addition to the retrospective analysis, prospective testing was performed on residual serum available at ARUP from patients tested for MOG antibodies at MCL. Specimens tested prospectively were obtained in one of three ways: as split aliquots prior to being sent to the MCL, as additional samples collected at the same time as the MCL sample, or as additional samples collected within 30 days of the original sample. These residual specimens were stored at −20°C until the time of testing. Each case was crosschecked with the retrospective analysis by the patient identification number and date of birth to avoid duplication of results in the final analysis. Additional samples from a validation cohort tested between October 2017 and February 2019 at MCL and used in validating the ARUP assay were included.

### ARUP laboratories MOG assay

Testing for antibodies to MOG using fCBA-IF was performed at ARUP Laboratories as recommended by the manufacturer (EUROIMMUN, FA 1156-1010-50 Anti-Myelin Oligodendrocyte Glycoprotein IIFT). Briefly, patient sera were screened at a 1:10 dilution on a substrate of fixed HEK293 cells transiently transfected to express a full-length human MOG protein. Slides were washed and incubated with FITC-conjugated anti-human IgG and examined for positivity by visual observation using a fluorescence microscope. If positive fluorescence was observed at the 1:10 dilution, additional testing was performed at serial 1:2 dilutions, and the highest dilution demonstrating positive fluorescence was reported as the end-point titer.

### Mayo clinical laboratory MOG assay

Testing for antibodies to MOG using live CBA-FC was performed at MCL as previously described (14). According to Mayo Clinical Laboratories reported protocols, patient sera are routinely screened at a 1:20 dilution on a substrate of live HEK293 cells expressing full-length MOG protein. Cells are washed and incubated with anti-human IgG1. Cells are washed again prior to evaluation by flow cytometry. Positivity is determined based on the ratio of mean fluorescence intensity (MFI) of transfected cells to MFI of non-transfected cells. A ratio of 2.5 or greater is considered positive. If samples screen positive at 1:20, additional testing is performed at 1:40, 1:100, and subsequent 10-fold dilutions (1,100, 1:10,000, etc.) until MFI ratio drops below 2.5.

### Statistical analysis

Correlation of parameters was analyzed with Spearman’s rank correlation coefficient. Cohen’s kappa statistic was used to assess the agreement between assays. All statistical analyses were performed using R Statistical Software (v4.1.2; R Core Team 2022) (21).

### Data availability

Anonymized data not published within this article will be made available by request from any qualified investigator.

## Results

**Figure 1:**
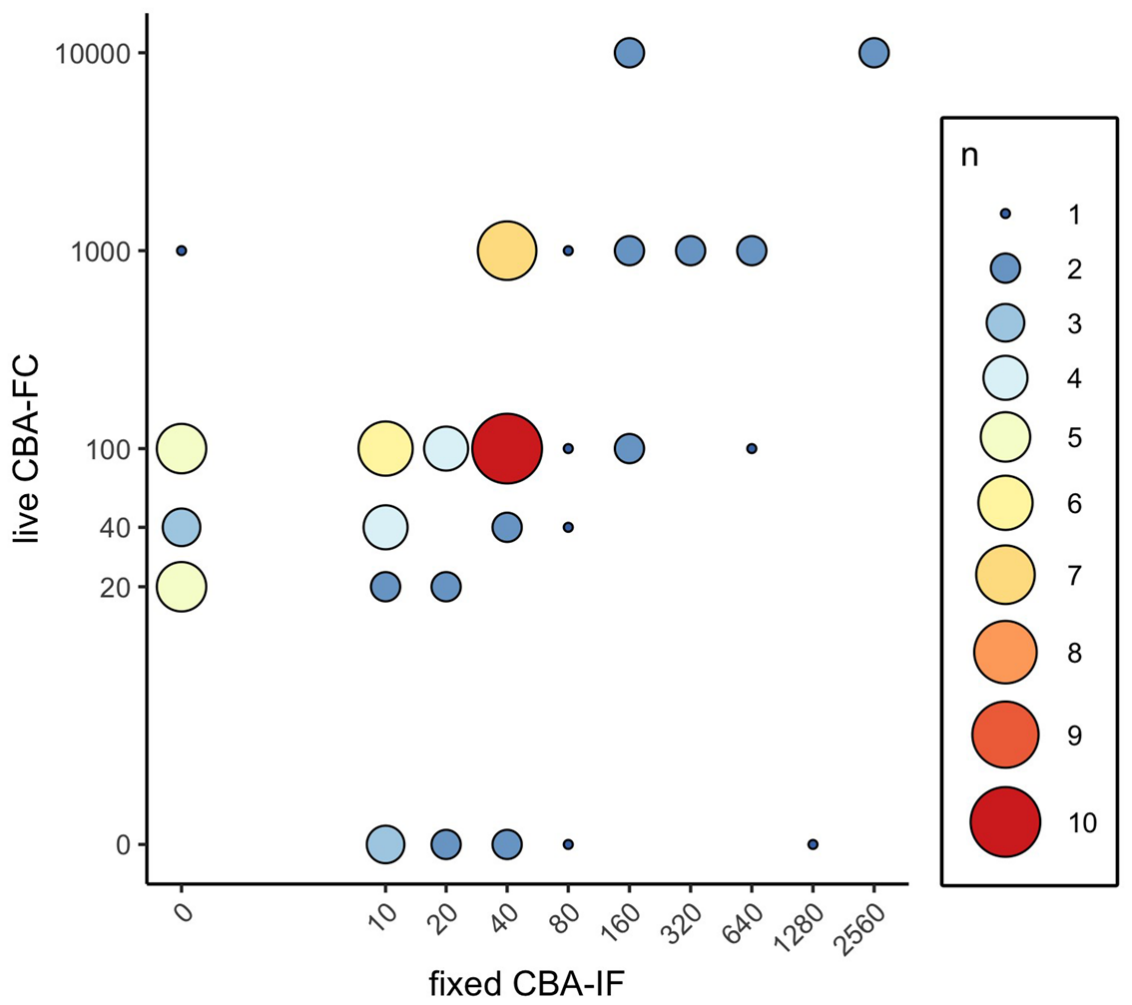
Distribution of MOG-IgG results by titer and frequency. Antibody titers of the live CBA-FC and fixed CBA-IF were plotted for all positive values. Size and color of plotted points represents the number of samples with the corresponding antibody titers.

In total, 322 serum samples were tested at both ARUP and MCL during the study period (Supplementary Figure 1). Of the 76 samples positive by either methodology, 53 were concordant between both assays, nine were positive by the ARUP fCBA-IF assay only, and 14 were positive by the MCL live CBA-FC assay only. 246 samples were negative by both assays. Overall, percent agreement between the two assays was 92.9 (Cohen’s kappa 0.78, [0.69–0.86]). Spearman correlation coefficient was 0.80 (*p* < 0.0001; Figure 1).

Of the 14 samples positive by live CBA-FC and negative by fCBA-IF, 8/14 (57%) had an antibody titer of ≤1:40, and 13/14 (93%) had an antibody titer of ≤1:100. Of the nine samples positive by fCBA-IF and negative by live CBA-FC, 7/9 (78%) had an antibody titer of ≤1:40 and 8/9 (89%) had an antibody titer of ≤1:80. Antibody titers are not identical between the live CBA-FC and fCBA-IF; of the 53 samples positive by both assays, 45/53 (85%) had a higher titer by live CBA-FC than by fCBA-IF.

## Discussion

In a set of 322 serum samples analyzed for MOG-IgG by both live CBA-FC and fCBA-IF at two different clinical reference labs, we found an overall agreement of 92.9%, with a strong Spearman correlation coefficient (0.80). Previous studies have compared live and fixed CBA using samples from clinically or serologically defined subsets of patients (18, 19). Specifically, Waters et al. performed a comparison between live CBA-FC, live CBA-IF, and fCBA-IF in a clinically defined group of patients with ADEM, seronegative NMO, optic neuritis, or longitudinally extensive transverse myelitis (*n* = 91) and found that of 25 samples positive by any of these methodologies, 21 were concordant on all three assays. The live CBA-IF detected 25 positives, the fCBA-IF detected 23 positives and the live CBA-FC detected 21 positives. In the control group of 244 MS patients, one false positive (FP) was identified by both the live CBA-FC and the fCBA-IF, and four additional FPs were identified by the fCBA-IF (18). Based on these results, the three assays have similar negative predictive values (ranging from 78.8 to 79.8) but diverging positive predictive value (100% for live CBA-IF, 95.5% for live CBA-FC, and 82.1% for fCBA-IF). While these statistics are valid, it is important to consider that this analysis includes a small number of discordant samples overall. Concluding that these data “emphasize the superiority of live CBA testing,” as some have suggested, is an oversimplification (22).

Reindl et al. (19) conducted an international study to compare 11 different assays for MOG antibodies at five different testing centers, using a strategy whereby a predefined set of positive or negative serum samples were tested across all platforms. Of note, predefined samples were determined based on testing via live CBA at four different institutions. Cell based assays tested in the study included seven live CBAs (four CBA-IF, three CBA-FC) and one fCBA-IF. Of the 39 clear positive samples tested, 32/39 were positive in all eight CBAs, and 36/39 were positive in all seven of the live CBAs. Of the 40 samples tested as clear negatives, 39/40 were negative in all eight CBAs, and 40/40 were negative in all seven live CBAs. Overall, there was 90% concordance between all the CBA (similar to the 92.9% seen in our study) and 96% concordance across the live CBAs. In a second phase of the same study, low positive and borderline negative samples were compared between the assay platforms; there was 77% agreement between all eight of the CBA platforms tested, without a clear distinction between live or fixed CBA being superior (19). The inclusion of these borderline negative and low positive samples highlights the limitations of all of these assays when evaluating borderline results, and the key role treating clinicians play in interpreting the results of these assays within the clinical context of each patient.

Positive cutoffs vary from assay to assay. The live CBA-FC reported here is the same one used in a study looking at the positive predictive value (PPV) of MOG testing at various assay cutoffs (23). In that paper, two neurologists reviewed the charts of 92 positive MOG-IgG1 assays and found 26/92 (28%) were designated as FP by both raters. When the end-titer of the assay for MOG was 1:20–40, the PPV was 51%; this increased to 82% at an end titer of 1:100 and 100% when the end-titer of ≥1:1,000 was used (23). To better understand the correlation between positive antibody titers in different assays, we plotted the assay titers for the live CBA-FC against the fCBA-IF in our study. Most of the samples that were negative by fCBA-IF yet positive by live CBA-FC had titers below 1:1,000 (Figure 1). This figure also illustrates that lower titers on fCBA-IF correspond to higher titers by live CBA-FC. As these assays have different methodologies, the titers are not directly comparable. This is important to consider based on the recently published International MOGAD Panel proposed diagnostic criteria, which recommends a cutoff of ≥1:100 for both of the assays described here to be considered a “clear positive,” with lower titers needing additional supporting clinical or MRI criteria to be considered consistent with MOGAD (12). Future studies applying these diagnostic criteria to patients tested by both live CBA-FC and fCBA-IF are needed to better understand the ideal cutoff for each individual assay to optimize sensitivity and specificity.

As a reference laboratory receiving samples from around the country, we did not have access to patient information in the discrepant cases to determine whether these incongruities represented false positives or false negatives in these assays. The absence of patient information in regard to the core clinical demyelinating event, supporting clinical or MRI features, and the temporal association of the tested sample with attack, relapse, or immunotherapy represents a clear limitation of this study. It is noteworthy that both the live CBA-FC and fCBA-IF identified some positives that the other assay did not, and that these discrepancies tended to occur at lower assay titers (see Figure 1). The preponderance of discrepancies at low titers reinforces the importance of applying clinical criteria in addition to antibody testing when making a diagnosis of MOGAD. Recognizing that different CBA testing methodologies will not give identical titers, and that higher titers by live CBA-FC generally correspond to lower titers by fCBA-IF is an important idiosyncrasy to be aware of when interpreting these results. Of the results positive by both assays in our comparison, 45/53 (85%) had a higher titer when measured by live CBA-FC than when tested by fCBA-IF.

Our study demonstrates that in a real-world reference laboratory setting, there is a high degree of agreement between fCBA-IF and live CBA-FC. This, along with data from prior studies comparing CBAs in clinically and serologically defined populations, confirms that both fixed and live CBA are a reasonable option for clinicians who suspect MOGAD in their patients and seek serologic confirmation. False positives and false negatives are a reality of all clinical laboratory testing; in the case of MOG, false positives may lead to treatment with an inappropriate immunosuppressive medication or delayed diagnosis of a different clinical entity. Avoiding indiscriminate testing for antineural antibodies and selecting tests based on clear clinical criteria is important to improve the positive predictive value of these assays. Clinicians need to consider testing availability, turnaround time, cost at their institution, and reliability when ordering any test. Future studies need to focus on improving testing reliability and determining markers of monophasic vs. relapsing disease to further inform treatment decisions.

## Data availability statement

The raw data supporting the conclusions of this article will be made available by the authors, without undue reservation.

## Author contributions

TS, TH, SC, and LP contributed to the conception and design of the study. TS, TH, and LZ organized the data. TS and LZ performed statistical analysis. KL assisted with data collection. TS wrote the first draft of the manuscript. TH, LZ, and LP wrote sections of the manuscript. All authors contributed to the article and approved the submitted version.

## Funding

This study was supported by the ARUP Institute for Clinical and Experimental Pathology. Fees for the publication of this manuscript were paid for by a generous contribution from The Sumaira Foundation.

## Conflict of interest

TS, TH, LZ, KL, and LP are affiliated with the ARUP Institute for Clinical and Experimental Pathology, which performs the fCBA-IF reported herein. They receive no royalties from the sale of myelin oligodendrocyte glycoprotein antibody testing; however, ARUP Laboratories receives revenue from conducting such tests.

The remaining author declares that the research was conducted in the absence of any commercial or financial relationships that could be construed as a potential conflict of interest.

## Publisher’s note

All claims expressed in this article are solely those of the authors and do not necessarily represent those of their affiliated organizations, or those of the publisher, the editors and the reviewers. Any product that may be evaluated in this article, or claim that may be made by its manufacturer, is not guaranteed or endorsed by the publisher.

## Supplementary material

Supplementary Figure S1 Flow chart showing consecutive steps for inclusion of sera in the study. **(A)** retrospective cohort strategy **(B)** prospective cohort strategy **(C)** validation cohort strategy.

## References

[1] Cobo-Calvo A Ruiz A Maillart E Audoin B Zephir H Bourre B. Clinical spectrum and prognostic value of CNS MOG autoimmunity in adults. Neurology. (2018) 90:e1858–69. doi: 10.1212/WNL.0000000000005560, PMID: 29695592

[2] Serin HM Yilmaz S Simsek E Kanmaz S Eraslan C Aktan G. Clinical spectrum, treatment and outcome of myelin oligodendrocyte glycoprotein (MOG) antibody-associated disease in children: a tertiary care experience. Acta Neurol Belg. (2021) 121:231–9. doi: 10.1007/s13760-020-01499-9, PMID: 33231843

[3] Wegener-Panzer A Cleaveland R Wendel EM Baumann M Bertolini A Häusler M. Clinical and imaging features of children with autoimmune encephalitis and MOG antibodies. Neurol Neuroimmunol Neuroinflamm. (2020) 7:e731. doi: 10.1212/NXI.0000000000000731, PMID: 32358225 PMC7217659

[4] De Mol C Wong Y Van Pelt E Wokke B Siepman T Neuteboom R. The clinical spectrum and incidence of anti-MOG-associated acquired demyelinating syndromes in children and adults. Mult Scler J. (2019) 26:806–14. doi: 10.1177/1352458519845112 PMC7294530 31094288

[5] Höftberger R Sepulveda M Armangue T Blanco Y Rostásy K Calvo AC. Antibodies to MOG and AQP4 in adults with neuromyelitis optica and suspected limited forms of the disease. Mult Scler J. (2015) 21:866–74. doi: 10.1177/1352458514555785, PMID: 25344373 PMC4824843

[6] Jarius S Ruprecht K Kleiter I Borisow N Asgari N Pitarokoili K. MOG-IgG in NMO and related disorders: a multicenter study of 50 patients. Part 2: epidemiology, clinical presentation, radiological and laboratory features, treatment responses, and long-term outcome. J Neuroinflammation. (2016) 13:280. doi: 10.1186/s12974-016-0718-0 27793206 PMC5086042

[7] Kitley J Woodhall M Waters P Leite MI Devenney E Craig J. Myelin-oligodendrocyte glycoprotein antibodies in adults with a neuromyelitis optica phenotype. Neurology. (2012) 79:1273–7. doi: 10.1212/WNL.0b013e31826aac4e, PMID: 22914827

[8] Kitley J Waters P Woodhall M Leite MI Murchison A George J. Neuromyelitis optica spectrum disorders with aquaporin-4 and myelin-oligodendrocyte glycoprotein antibodies: a comparative study. JAMA Neurol. (2014) 71:276–83. doi: 10.1001/jamaneurol.2013.5857, PMID: 24425068

[9] Sato DK Callegaro D Lana-Peixoto MA Waters PJ Jorge FMH Takahashi T. Distinction between MOG antibody-positive and AQP4 antibody-positive NMO spectrum disorders. Neurology. (2014) 82:474–81. doi: 10.1212/WNL.0000000000000101, PMID: 24415568 PMC3937859

[10] Pröbstel AK Rudolf G Dornmair K Collongues N Chanson JB Sanderson NS. Anti-MOG antibodies are present in a subgroup of patients with a neuromyelitis optica phenotype. J Neuroinflammation. (2015) 12:46. doi: 10.1186/s12974-015-0256-1 25889963 PMC4359547

[11] Jarius S Paul F Aktas O Asgari N Dale RC De SJ. MOG encephalomyelitis: international recommendations on diagnosis and antibody testing. J Neuroinflammation. (2018) 15:134. doi: 10.1186/s12974-018-1144-2 29724224 PMC5932838

[12] Banwell B Bennett JL Marignier R Kim HJ Brilot F Flanagan EP. Diagnosis of myelin oligodendrocyte glycoprotein antibody-associated disease: international MOGAD panel proposed criteria. Lancet Neurol. (2023) 22:268–82. doi: 10.1016/S1474-4422(22)00431-8, PMID: 36706773

[13] Reindl M Linington C Brehm U Egg R Dilitz E Deisenhammer F. Antibodies against the myelin oligodendrocyte glycoprotein and the myelin basic protein in multiple sclerosis and other neurological diseases: a comparative study. Brain. (1999) 122:2047–56. doi: 10.1093/brain/122.11.2047 10545390

[14] Waters P Woodhall M O’Connor KC Reindl M Lang B Sato DK. MOG cell-based assay detects non-MS patients with inflammatory neurologic disease. Neurol Neuroimmunol Neuroinflammation. (2015) 2:e89. doi: 10.1212/NXI.0000000000000089, PMID: 25821844 PMC4370386

[15] Cobo-Calvo Á d’Indy H Ruiz A Collongues N Kremer L Durand-Dubief F. Frequency of myelin oligodendrocyte glycoprotein antibody in multiple sclerosis: a multicenter cross-sectional study. Neurol Neuroimmunol Neuroinflammation. (2020) 7:e649. doi: 10.1212/NXI.0000000000000649, PMID: 31836640 PMC6943364

[16] Tea F Lopez JA Ramanathan S Merheb V Lee FXZ Zou A. Characterization of the human myelin oligodendrocyte glycoprotein antibody response in demyelination. Acta Neuropathol Commun. (2019) 7:145. doi: 10.1186/s40478-019-0786-3 31481127 PMC6724269

[17] Pauli FD Berger T. Myelin oligodendrocyte glycoprotein antibody-associated disorders: toward a new spectrum of inflammatory demyelinating CNS disorders? Front Immunol. (2018) 9:2753. doi: 10.3389/fimmu.2018.02753 30555462 PMC6281762

[18] Waters PJ Komorowski L Woodhall M Lederer S Majed M Fryer J. A multicenter comparison of MOG-IgG cell-based assays. Neurology. (2019) 92:e1250–5. doi: 10.1212/WNL.0000000000007096, PMID: 30728305 PMC6511109

[19] Reindl M Schanda K Woodhall M Tea F Ramanathan S Sagen J. International multicenter examination of MOG antibody assays. Neurol Neuroimmunol Neuroinflamm. (2020) 7:e674. doi: 10.1212/NXI.0000000000000674, PMID: 32024795 PMC7051197

[20] Gastaldi M Scaranzin S Jarius S Wildeman B Zardini E Mallucci G. Cell-based assays for the detection of MOG antibodies: a comparative study. J Neurol. (2020) 267:3555–64. doi: 10.1007/s00415-020-10024-0, PMID: 32623596

[21] R Core Team. R: A language and environment for statistical computing. R Foundation for Statistical Computing: Vienna, Austria. (2013). Available at: http://www.R-project.org/

[22] Yeh EA Nakashima I. Live-cell based assays are the gold standard for anti-MOG-ab testing. Neurology. (2019) 92:501–2. doi: 10.1212/WNL.0000000000007077, PMID: 30728306

[23] Sechi E Buciuc M Pittock SJ Chen JJ Fryer JP Jenkins SM. Positive predictive value of myelin oligodendrocyte glycoprotein autoantibody testing. JAMA Neurol. (2021) 78:741–6. doi: 10.1001/jamaneurol.2021.0912, PMID: 33900394 PMC8077043

