# Supplementary figures and images for: High level of agreement in a fixed vs. live cell-based assay for antibodies to myelin oligodendrocyte glycoprotein in a real-world clinical laboratory setting
Source: Front Neurol. 2023 Jul 12;14:1192644. doi: 10.3389/fneur.2023.1192644 (PMC10368875; doi:10.3389/fneur.2023.1192644)

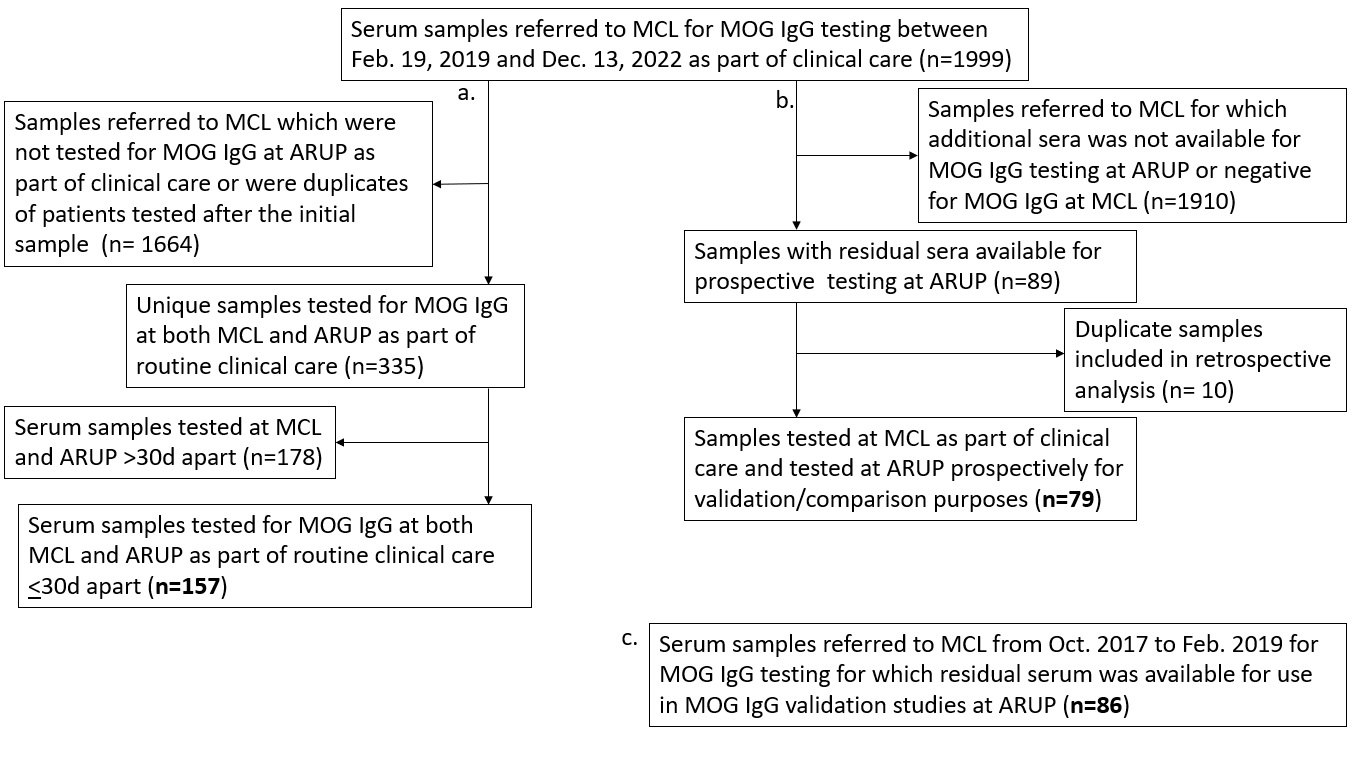

Supplement: Supplementary Figure S1 — Flow chart showing consecutive steps for inclusion of sera in the study. (A) retrospective cohort strategy (B) prospective cohort strategy (C) validation cohort strategy. [file Image_1.JPEG]
